# Supplementary material for: Development of SSR Markers Linked to Stress Responsive Genes along Tomato Chromosome 3 (Solanum lycopersicum L.)
Source: BioTech (Basel). 2022 Aug 16;11(3):34. doi: 10.3390/biotech11030034 (PMC9397033; doi:10.3390/biotech11030034)
Supplement: Supplementary file 1 [file biotech-11-00034-s001.zip › biotech-1839836-supplementary.pdf]

BioTech

Development of SSR markers linked to stress responsive genes along tomato chromosome 3 (*Solanum lycopersicum* L.)

Mohammad Brake, Lana Al-Qadumii, Hassan Hamasha, Hussein Migdadi, Abi Awad, Nizar Haddad and Monther T. Sadder

Supplementary Table S1. Nearby genes for developed 20 SSR markers along with their description.

SSR locus	Gene locus	Chromosome coordinates	Description
ju003		SL2.50ch03: 67710651.. 67710885	
	Solyc03g118960	SL2.50ch03:67708555..67710621	Multidrug and toxin extrusion protein 1 (AHRD V1 *--- S47A1_XENTR); contains Interpro domain(s) IPR015521 MATE family transporter related protein
	Solyc03g118970	SL2.50ch03:67712885..67715799	Multidrug resistance protein mdtK (AHRD V1 ***- MDTK_CITK8); contains Interpro domain(s) IPR015521 MATE family transporter related protein
	Solyc03g118950	SL2.50ch03:67706003..67707289	DsRNA-binding protein 3 (AHRD V1 ***- D7LRB5_ARALY); contains Interpro domain(s) IPR001159 Double-stranded RNA binding
ju004		SL2.50ch03: 64521427..64521653	
	Solyc03g114580	SL2.50ch03:64520115..64530361	Uridine kinase (AHRD V1 *-*- C3SA58_BRADI); contains Interpro domain(s) IPR000764 Uridine kinase
	Solyc03g114560	SL2.50ch03:64512634..64513982	Strictosidine synthase family protein (AHRD V1 ***- D7LVM7_ARALY); contains Interpro domain(s) IPR004141 Strictosidine synthase
	Solyc03g114550	SL2.50ch03:64510484..64511709	Strictosidine synthase family protein (AHRD V1 ***- D7LVM7_ARALY); contains Interpro domain(s) IPR004141 Strictosidine synthase
ju006		SL2.50ch10: 42685545.. 42685785	
	Solyc10g047820	SL2.50ch10:42763906..42766189	Transporter CorA family (AHRD V1 *-*- Q62C47_BURMA)
	Solyc10g047810	SL2.50ch10:42751106..42753214	Receptor protein kinase-like protein (AHRD V1 ***- Q9LSL5_ARATH); contains Interpro domain(s) IPR002290 Serine/threonine protein kinase
	Solyc10g047780	SL2.50ch10:42473191..42474359	Seed lectin (AHRD V1 *-*- LECS_VATMA); contains Interpro domain(s) IPR001220 Legume lectin, beta chain
	Solyc10g047770	SL2.50ch10:42457293..42457550	Receptor protein kinase-like protein (AHRD V1 ***- Q9LSL5_ARATH); contains Interpro domain(s) IPR017442 Serine/threonine protein kinase-related
ju007		SL2.50ch03: 66570341.. 66570470	
	Solyc03g117370	SL2.50ch03:66533935..66538545	WD-40 repeat protein (AHRD V1 *--- B2J4D1_NOSP7); contains Interpro domain(s) IPR017986 WD40 repeat, region
	Solyc03g117420	SL2.50ch03:66554722..66569270	Leucine-rich repeat family protein (AHRD V1 ***- D7KDI8_ARALY); contains Interpro domain(s) IPR006553 Leucine-rich repeat, cysteine-containing subtype
	Solyc03g117380	SL2.50ch03:66539750..66540396	Topless-related protein 1 (AHRD V1 -**- TPR1_ARATH)
	Solyc03g117470	SL2.50ch03:66615377..66620417	Calcineurin subunit B (AHRD V1 *-*- B6U4N5_MAIZE); contains Interpro domain(s) IPR011992 EF-Hand type
	Solyc03g117480	SL2.50ch03:66624199..66629058	Pyrophosphate-energized proton pump (Pyrophosphate-energized inorganic pyrophosphatase) (H(+)-PPase) (AHRD V1 **** C7HW54_9FIRM); contains Interpro domain(s) IPR004131 Inorganic H+ pyrophosphatase

	Solyc03g117450	SL2.50ch03:66611609..66612183	Major allergen Mal d 1 (AHRD V1 ***- Q43550_MALDO); contains Interpro domain(s) IPR000916 Bet v I allergen
	Solyc03g117430	SL2.50ch03:66599928..66607724	Cobalamin synthesis protein P (AHRD V1 ***- D3M8Z8_9ACTO); contains Interpro domain(s) IPR003495 Cobalamin (vitamin B12) biosynthesis CobW-like
ju008		SL2.50ch03:53049368..53049607	
	Solyc03g083200	SL2.50ch03:53043734..53044215	structural maintenance of chromosomes flexible hinge domain-containing protein GMI1 isoform X11 [
	Solyc03g083190	SL2.50ch03:53030731..53037929	structural maintenance of chromosomes flexible hinge domain-containing protein GMI1 isoform X3
	Solyc03g083210	SL2.50ch03:53049663..53064618	At5g24280 (Fragment) (AHRD V1 *-*- Q3YIX0_ARATH)
	Solyc03g083220	SL2.50ch03:53071328..53072381	Helicase-like protein (AHRD V1 *-*- Q69V29_ORYSJ); contains Interpro domain(s) IPR010285 Protein of unknown function DUF889, eukaryote
ju010		SL2.50ch03: 3324789.. 3324994	
	Solyc03g026010	SL2.50ch03:3420662..3422038	Transmembrane protein 161B (AHRD V1 *-*- T161B_MOUSE)
	Solyc03g025970	SL2.50ch03:3394813..3400693	Methyl binding domain protein (AHRD V1 *-*- B9I4C0_POPTR); contains Interpro domain(s) IPR001739 Methyl-CpG DNA binding
	Solyc03g025980	SL2.50ch03:3402975..3407759	Peptidyl-prolyl cis-trans isomerase (Fragment) (AHRD V1 ***- A9Y0F6_9MYRT); contains Interpro domain(s) IPR007062 Protein phosphatase inhibitor 2 (IPP-2)
	Solyc03g025950	SL2.50ch03:3384122..3387029	Membrane-associated progesterone receptor component 1 (AHRD V1 ***- C0NU44_AJECG); contains Interpro domain(s) IPR001199 Cytochrome b5
	Solyc03g025920	SL2.50ch03:3334446..3335904	F-box family protein (AHRD V1 ***- D7LS19_ARALY); contains Interpro domain(s) IPR001810 Cyclin-like F-box
	Solyc03g025870	SL2.50ch03:3256762..3259518	MYB transcription factor (AHRD V1 **-* Q9LTJ5_ARATH); contains Interpro domain(s) IPR015495 Myb transcription factor
	Solyc03g025840	SL2.50ch03:3238954..3241512	Cytochrome b561 (AHRD V1 ***- Q3LGX5_CITLA); contains Interpro domain(s) IPR004877 Cytochrome b561, eukaryote
ju011		SL2.50ch03:70061829..70062070	
	Solyc03g122100	SL2.50ch03:70060756..70065647	3-oxoacyl-synthase I (AHRD V1 *-*- B6SJZ0_MAIZE); contains Interpro domain(s) IPR016038 Thiolase-like, subgroup
	Solyc03g122080	SL2.50ch03:70050315..70052922	Legume lectin beta domain (AHRD V1 *-*- A2Q3C0_MEDTR); contains Interpro domain(s) IPR010341 Protein of unknown function DUF936, plant
	Solyc03g122040	SL2.50ch03:70031953..70041568	COP1-Interacting ProteinI 7 (CIP7) (Fragment) (AHRD V1 *--- Q7GDB1_ARATH)
	Solyc03g122090	SL2.50ch03:70055292..70058642	Protein transport protein Sec22 (AHRD V1 **-- B0CRV5_LACBS); contains Interpro domain(s) IPR001388 Synaptobrevin IPR010908 Longin
	Solyc03g122070	SL2.50ch03:70047510..70049605	Uncharacterized ABC transporter ATP-binding protein/permease C9B6.09c (AHRD V1 **-- YNT9_SCHPO); contains Interpro domain(s) IPR003439 ABC transporter-like
	Solyc03g122130	SL2.50ch03:70079537..70082001	L-lactate dehydrogenase (AHRD V1 ***- C0S8Q7_PARBP); contains Interpro domain(s) IPR012133 Alpha-hydroxy acid dehydrogenase, FMN-dependent
ju014		SL2.50ch03:66799080..66799311	

	Solyc03g117750	SL2.50ch03:66789853..66794315	Polygalacturonase (AHRD V1 **-- B6SZN5_MAIZE); contains Interpro domain(s) IPR012334 P
	Solyc03g117730	SL2.50ch03:66779750..66783039	Tubby-like protein 13 (AHRD V1 ***- D2CMM7_ORYSJ); contains Interpro domain(s) IPR000007 Tubby, C-terminal
	Solyc03g117720	SL2.50ch03:66775127..66779386	AP2-like ethylene-responsive transcription factor At1g16060 (AHRD V1 ***- AP2L1_ARATH); contains Interpro domain(s) IPR001471 Pathogenesis-related transcriptional factor and ERF, DNA-binding
	Solyc03g117770	SL2.50ch03:66801297..66806435	Serine incorporator 1 (AHRD V1 **-* B5X3W1_SALSA); contains Interpro domain(s) IPR005016 TMS membrane protein/tumour differentially expressed protein
	Solyc03g117740	SL2.50ch03:66786743..66789552	Chloroplast At1g16080 protein (Fragment) (AHRD V1 *-*- Q1HRY1_SOLLC)
	Solyc03g117790	SL2.50ch03:66813605..66820539	Serine/threonine protein kinase (AHRD V1 *-*- Q16PN5_AEDAE); contains Interpro domain(s) IPR002290 Serine/threonine protein kinase
	Solyc03g117780	SL2.50ch03:66806776..66811545	UV excision repair protein RAD23 (AHRD V1 ***- B6JX15_SCHJY); contains Interpro domain(s) IPR004806 UV excision repair protein Rad23
ju015		SL2.50ch03: 4614781..4615028	
	Solyc03g032020	SL2.50ch03:4516620..4519322	Hydroxy-methylglutaryl-coenzyme A reductase (AHRD V1 **** O48624_TOBAC); contains Interpro domain(s) IPR002202 Hydroxymethylglu-taryl-CoA reductase, class I/II, catalytic
	Solyc03g032070	SL2.50ch03:4583223..4586185	Holliday junction ATP-dependent DNA helicase ruvB (AHRD V1 **** D0N0A1_PHYIN); contains Interpro domain(s) IPR010339 TIP49, C-terminal
	Solyc03g032060	SL2.50ch03:4571144..4571680	Ring finger protein (AHRD V1 *-*- O49869_HORVU); contains Interpro do-main(s) IPR018957 Zinc finger, C3HC4 RING-type
	Solyc03g032040	SL2.50ch03:4555538..4559925	MFS family major facilitator transporter D-xylose cation symporter (AHRD V1 *-** D4E1S5_SEROD); contains Interpro domain(s) IPR016196 Major facilitator superfamily, general substrate transporter
	Solyc03g032090	SL2.50ch03:4609738..4611021	Amino acid transporter family protein (AHRD V1 **** D7LI10_ARALY); con-tains Interpro domain(s) IPR013057 Amino acid transporter, transmembrane
	Solyc03g032150	SL2.50ch03:4653491..4658660	Serine/threonine kinase-like protein ABC1063 (AHRD V1 **** Q1KMP8_HORVD); contains Interpro domain(s) IPR002290 Serine/threonine protein kinase
ju017		SL2.50ch03:67271681..67271951	
	Solyc03g118380	SL2.50ch03:67272508..67272981	DNA-directed RNA polymerase II subunit (AHRD V1 **** A8N5F1_COPC7); contains Interpro domain(s) IPR012340 Nucleic acid-binding, OB-fold
	Solyc03g118370	SL2.50ch03:67265834..67269224	Serine carboxypeptidase K10B2.2 (AHRD V1 **** B6TKD2_MAIZE); contains Interpro domain(s) IPR001563 Peptidase S10, serine carboxypeptidase
	Solyc03g118390	SL2.50ch03:67278298..67287556	Coiled-coil domain-containing protein SCD2
ju022		SL2.50ch12:5685216.. 5685492	
	Solyc12g014630	SL2.50ch12:5630952..5631575	Cortical cell-delineating protein (AHRD V1 *--- B6U436_MAIZE); contains Interpro domain(s) IPR013770 Plant lipid transfer protein and hydrophobic protein, helical
	Solyc12g015640	SL2.50ch12:5642779..5645448	Mitochondrial transcription termination factor (Fragment) (AHRD V1 *--- C6FB12_PSEMZ); contains Interpro domain(s) IPR003690 Mitochodrial tran-

			scription termination factor-related
	Solyc12g015650	SL2.50ch12:5649944..5651353	Armadillo repeat containing 4 (AHRD V1 **-- A8K906_HUMAN); contains Interpro domain(s) IPR011989 Armadillo-like helical
	Solyc12g015660	SL2.50ch12:5655855..5659081	Zinc ion binding protein (AHRD V1 **-- D7KN38_ARALY); contains Interpro domain(s) IPR013536 WLM
	Solyc12g015680	SL2.50ch12:5664926..5665519	Ycf60 protein (AHRD V1 **-- Q8DJZ7_THEEB)
	Solyc12g015690	SL2.50ch12:5671093..5671896	Fasciclin-like arabinogalactan protein 10 (AHRD V1 ***- A9XTL5_GOSHI); contains Interpro domain(s) IPR000782 FAS1 domain
ju023		SL2.50ch12:5579349.. 5579659	
	Solyc12g014590	SL2.50ch12:5578789..5581766	Pirin (AHRD V1 ***- D7RJ70_CARPA); contains Interpro domain(s) IPR012093 Pirin
	Solyc12g014580	SL2.50ch12:5577417..5577847	Pollen allergen Ole e 6 (AHRD V1 *-*- ALL6_OLEEU); contains Interpro domain(s) IPR015333 Pollen allergen ole e 6
	Solyc12g014570	SL2.50ch12:5569788..5576277	Glycerophosphoryl diester phosphodiesterase family protein (AHRD V1 ***- D7MKI0_ARALY); contains Interpro domain(s) IPR017946 PLC-like phosphodiesterase, TIM beta/alpha-barrel domain
	Solyc12g014600	SL2.50ch12:5600467..5603988	Heterogeneous nuclear ribonucleoprotein A3 (AHRD V1 *--- B6TGB9_MAIZE); contains Interpro domain(s) IPR000504 RNA recognition motif, RNP-1
	Solyc12g014660	SL2.50ch12:5655855..5659081	Zinc ion binding protein (AHRD V1 **-- D7KN38_ARALY); contains Interpro domain(s) IPR013536 WLM
	Solyc12g014610	SL2.50ch12:5610112..5616122	WRKY transcription factor 78 (AHRD V1 ***- Q5HZ67_ORYSA); contains Interpro domain(s) IPR003657 DNA-binding WRKY
ju026		SL2.50ch03:2747742.. 2747972	
	Solyc03g025310	SL2.50ch03:2751529..2759474	Sec-independent protein translocase protein tatA/E homolog (AHRD V1 *-*- D4TUR0_9NOST); contains Interpro domain(s) IPR006312 Twin-arginine translocation protein TatA/E
	Solyc03g025300	SL2.50ch03:2743304..2743489	protein FAR1-RELATED SEQUENCE 6-like
	Solyc03g025290	SL2.50ch03:2734190..2738566	Hydroxyproline-rich glycoprotein family protein
	Solyc03g025320	SL2.50ch03:2760932..2764000	Hydroxycinnamoyl transferase (AHRD V1 **-* D2XJ64_9MAGN); contains Interpro domain(s) IPR003480 Transferase
ju027		SL2.50ch03:2948914..2949151	
	Solyc03g025530	SL2.50ch03:2911517..2919038	S-layer domain protein (AHRD V1 *-*- B2J947_NOSP7); contains Interpro domain(s) IPR001119 S-layer homology region
	Solyc03g025510	SL2.50ch03:2898281..2903218	Calmodulin-binding heat shock protein (AHRD V1 **-* Q1RN86_GOSHI); contains Interpro domain(s) IPR002921 Lipase, class 3
	Solyc03g025500	SL2.50ch03:2893357..2896872	Prolyl 4-hydroxylase alpha subunit-like protein (AHRD V1 ***- Q9LSI6_ARATH); contains Interpro domain(s) IPR006620 Prolyl 4-hydroxylase, alpha subunit
	Solyc03g025550	SL2.50ch03:2943318..2949033	Signal peptide peptidase family protein (AHRD V1 ***- D7M8R6_ARALY); contains Interpro domain(s) IPR007369 Peptidase A22B, signal peptide peptidase
	Solyc03g025560	SL2.50ch03:2960198..2962401	Undecaprenyl pyrophosphate synthase (AHRD V1 **** UPPS_PYRFU); contains

	Solyc03g025600	SL2.50ch03:2995919..3004569	Interpro domain(s) IPR001441 Di-trans-poly-cis-decaprenylcistransferase-like Pectinacetylesterase like protein (Fragment) (AHRD V1 *--- Q56WP8_ARATH); contains Interpro domain(s) IPR004963 Pectinacetylesterase
ju029		SL2.50ch03:69455523.. 69455806	
	Solyc03g121270	SL2.50ch03:69429833..69434237	IAA-amino acid hydrolase (AHRD V1 **** D5FTH2_POPTO); contains Interpro domain(s) IPR017439 Peptidase M20D, mername-AA028/carboxypeptidase Ss1
	Solyc03g121290	SL2.50ch03:69441939..69444677	F-box family protein (AHRD V1 ***- B9I3S8_POPTR); contains Interpro domain(s) IPR001810 Cyclin-like F-box
	Solyc03g121310	SL2.50ch03:69446948..69451780	RWD domain-containing protein (AHRD V1 ***- Q38HU0_SOLTU); contains Interpro domain(s) IPR006575 RWD
	Solyc03g121350	SL2.50ch03:69465476..69471549	Anoctamin-like protein (AHRD V1 ***- D0MXY8_PHYIN); contains Interpro domain(s) IPR007632 Protein of unknown function DUF590
	Solyc03g121360	SL2.50ch03:69474092..69474703	Charged multivesicular body protein 1b (AHRD V1 ***- B6T9W6_MAIZE); contains Interpro domain(s) IPR005024 Snf7
ju035		SL2.50ch03:63216535.. 63216811	
	Solyc03g112890	SL2.50ch03:63173294..63175404	Myb family transcription factor (AHRD V1 *-*- D7M9F3_ARALY); contains Interpro domain(s) IPR015495 Myb transcription factor
	Solyc03g112910	SL2.50ch03:63189344..63204073	Pantothenate kinase (AHRD V1 *-** C1FDA4_9CHLO); contains Interpro domain(s) IPR015844 Pantothenate kinase, acetyl-CoA regulated, two-domain type
	Solyc03g112900	SL2.50ch03:63185027..63188415	Iron-sulfur cluster assembly scaffold protein IscU (AHRD V1 **** D0I7Q6_VIBHO); contains Interpro domain(s) IPR002871 NIF system FeS cluster assembly, NifU, N-terminal
	Solyc03g112920	SL2.50ch03:63207372..63207902	C2H2L domain class transcription factor (AHRD V1 ***- D9ZIU3_MALDO)
	Solyc03g112930	SL2.50ch03:63221882..63223966	Dof zinc finger protein-like (AHRD V1 ***- Q0WPR9_ARATH); contains Interpro domain(s) IPR003851 Zinc finger, Dof-type
	Solyc03g112950	SL2.50ch03:63256327..63287724	Serine/threonine-protein kinase (AHRD V1 ***- D2V0W7_NAEGR); contains Interpro domain(s) IPR015519 Ataxia-Telangiectasia Mutated
ju037		SL2.50ch03:64862699..64862925	
	Solyc03g114980	SL2.50ch03:64837867..64839736	Unknown Protein (AHRD V1)
	Solyc03g115000	SL2.50ch03:64852540..64857706	Belongs to the 26 member TON1 Recruiting Motif family. Interacts with OFPs through the M8 motif.
	Solyc03g115010	SL2.50ch03:64866814..64867839	Transcription factor CYCLOIDEA (Fragment) (AHRD V1 *-*- CYCLD_ANTML); contains Interpro domain(s) IPR005333 Transcription factor, TCP
	Solyc03g115030	SL2.50ch03:64880092..64882233	Mitochondrial import receptor subunit TOM5 homolog (AHRD V1 ***- TOM5_ARATH)
	Solyc03g115040	SL2.50ch03:64882651..64884482	Xylanase inhibitor (Fragment) (AHRD V1 **-- Q53IQ4_WHEAT); contains Interpro domain(s) IPR001461 Peptidase A1
ju039		SL2.50ch04: 25472989..25473278	
	Solyc04g025160	SL2.50ch04:25432546..25438502	Arsenite ATPase transporter (Eurofung) (AHRD V1 **** C8VJ74_EMENI); contains Interpro domain(s) IPR003348 ATPase, anion-transporting
	Solyc04g025120	SL2.50ch04:25460162..25460317	Arsenite translocating ATPase-like protein (Fragment) (AHRD V1 ***-

			A8JGB0_CHLRE)
ju040		SL2.50ch03:65907225..65907561	
	Solyc03g116500	SL2.50ch03:65900461..65904769	polygalacturonase (XOPG1)
	Solyc03g116530	SL2.50ch03:65913029..65914334	Glyoxalase/bleomycin resistance protein/dioxygenase (AHRD V1 ***- Q2HVV5_MEDTR); contains Interpro domain(s) IPR004360 Glyoxalase/bleomycin resistance protein/dioxygenase
ju041		SL2.50ch03:18327688..18328001	
	Solyc03g071690	SL2.50ch03:18328472..18330381	Non-symbiotic hemoglobin 2 (AHRD V1 **-- D7L8Z8_ARALY); contains Interpro domain(s) IPR001032 Leghaemoglobin
	Solyc03g071680	SL2.50ch03:18320038..18322555	golgin subfamily A member 6-like protein 22 isoform X1
	Solyc03g071670	SL2.50ch03:18316096..18318175	Protein binding protein (AHRD V1 ***- D7LXJ1_ARALY)
	Solyc03g071660	SL2.50ch03:18305959..18306838	WD40 repeat domain-containing protein (AHRD V1 **-- D2V3E5_NAEGR)
